# Beach volleyball and Cutaneous Larva Migrans

**DOI:** 10.1093/jtm/taad087

**Published:** 2023-06-27

**Authors:** Anna Kuna, Romuald Olszański, Agnieszka Wroczyńska, Beata Biernat, Katarzyna Sikorska

**Affiliations:** Department of Tropical and Parasitic Diseases, Institute of Maritime and Tropical Medicine, Faculty of Health Sciences, Medical University of Gdansk, Poland; University Center for Maritime and Tropical Medicine; University Center for Maritime and Tropical Medicine, Outpatient Clinic; Department of Tropical Parasitology, Institute of Maritime and Tropical Medicine, Faculty of Health Sciences, Medical University of Gdansk; Division of Tropical Medicine and Epidemiology, Institute of Maritime and Tropical Medicine, Faculty of Health Sciences, Medical University of Gdansk, Poland

**Keywords:** Parasite, pruritus, cutaneous larva migrans, CLM, tropics, skin

## Abstract

Cutaneous larva migrans can affect even athletes who travelled to play beach volleyball in Zanzibar. We describe a cluster of CLM infections in travellers who contracted the disease during their trip to Africa, rather than bringing a volleyball trophy. Despite presenting typical changes, all of them were misdiagnosed.

Cutaneous Larva Migrans (CLM) is a tropical dermatosis^1^ caused by hookworms belonging mainly to *Ancylostoma* and *Uncinaria* spp. CLM is mainly caused by hookworm larvae present in contaminated with animal faeces soil. After direct contact with human skin, hookworm larvae enter dermis causing the disease.^2^ In the case of CLM of zoonotic origin, the larvae can only localize in the human skin due to the lack of collagenase, which enables the parasite to migrate deeper into the host’s tissues.^3^ Humans are accidental hosts, and the incubation period for the disease lasts between 5 and 15 days.^1^ The disease typically occurs in tourists walking barefoot or sunbathing on contaminated beaches.

We describe a case of 12 travellers with CLM, reporting skin changes on their feet after a 14 days tourist stay in Zanzibar. The group consisted of 20 people, aged from 30 to 35 years, with no significant medical history, who were amateur beach volleyball players and took a part in local tournaments of this game. During their stay, the group played beach volleyball barefoot on a public beach and relaxed there. Skin changes began to appear in subsequent travellers towards the end of their stay, during their return journey and after arriving home.

The initial symptom for all patients was redness and a pruritic blister on the skin of the foot. Later, serpiginous, erythematous, elevated lesions up to several centimetres in length with a papule or vesicle at the end appeared (Figs 1–3). The itching was most intense at night.

**Figure 1:**
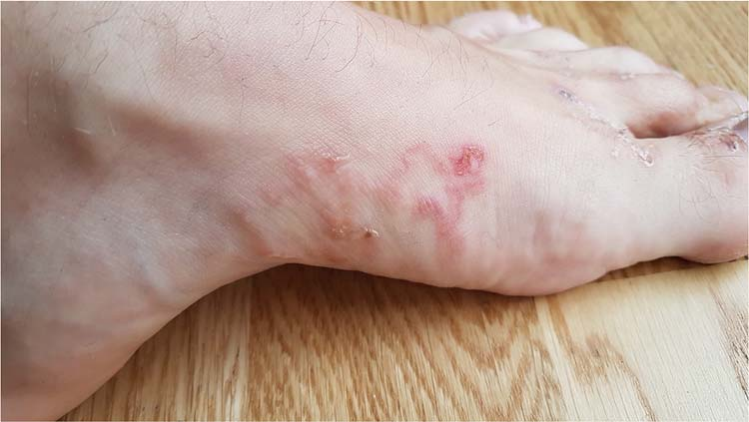
CLM presentation in Patient 1.

**Figure 2:**
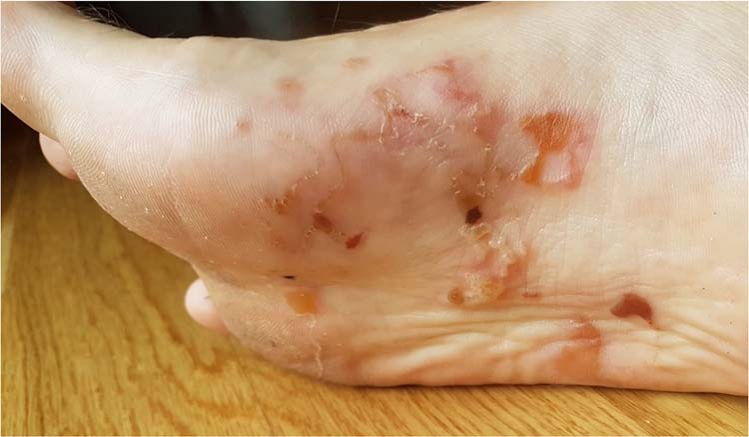
CLM presentation in Patient 2.

**Figure 3:**
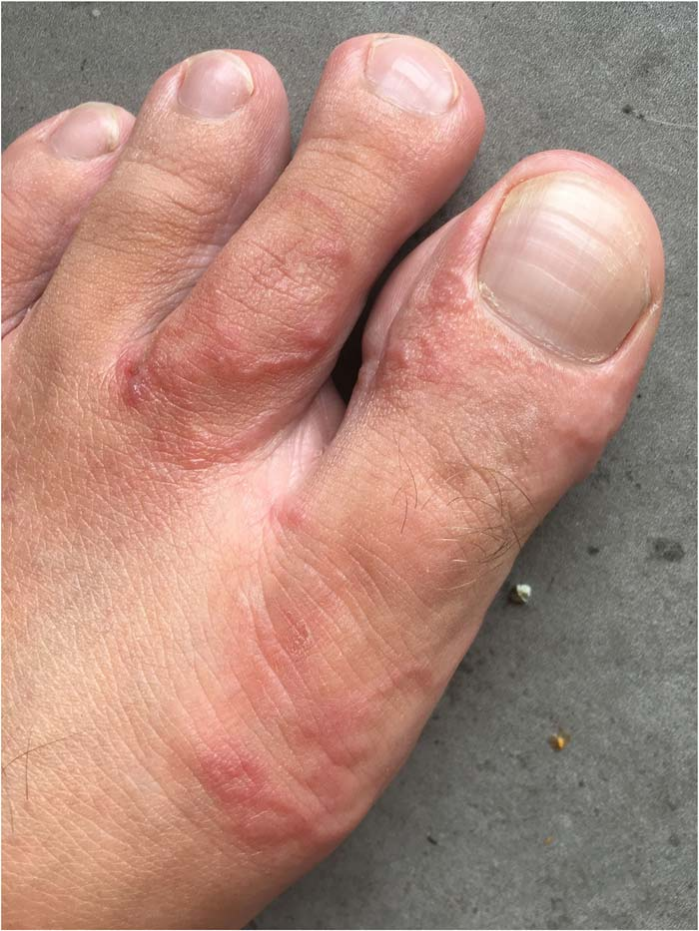
CLM presentation in Patient 3.

Patients sought help from various doctors for several days, who misdiagnosed their condition as urticaria, contact dermatitis, fungal infections and atopic dermatitis. They were given ineffective medications, including topical and oral antibiotics, antifungals and corticosteroids.

Finally, the patients were diagnosed with CLM and treated with oral albendazole, ivermectin as a cream and cryotherapy once in the area of the lesion. The itching disappeared in all patients within 1–3 days, and complete regression of skin lesions was observed after 15–21 days with post-inflammatory hyperpigmentation.

CLM is a common diagnosis among skin problems after travelling to tropical areas. Cutaneous changes are diagnosed among ill travellers as one of the top three most common conditions.^4^ The image of CLM invasion is so specific that seeing this disease once is enough to remember it for a lifetime. Preventing CLM infection involves avoiding direct skin contact with soil that may be contaminated with animal faeces. Despite the well-known epidemiology of CLM and its characteristic symptoms, difficulties in obtaining an accurate diagnosis by patients still persist. In 2021, a similar cluster of CLM infections was described, but diagnoses were established among consecutive patients admitted to the hospital who had returned from Zanzibar.^5^ In our study, we describe cases within one group of travellers, with 12 out of 20 participants contracted the infection. They were diagnosed and treated on an outpatient basis at the dermatology clinic. Therefore, promoting medical knowledge about imported diseases is still necessary due to the increasing number of travellers and the risk of importing infections from tropical regions. It is worth noting that cryotherapy is currently not recommended in the expert guidelines, similarly to topical ivermectin, both mentioned above. The first-line treatment should remain oral ivermectin or albendazole.

## Data Availability

Data availability statement are available from authors upon request.

## CRediT author statement

Anna Kuna (Conceptualization-Lead, Data curation-Lead, Formal analysis-Lead, Investigation-Lead, Project administration-Lead, Resources-Lead, Supervision-Lead, Visualization-Lead, Writing—original draft-Lead, Writing—review and editing-Lead), Romuald Olszański (Conceptualization-Supporting, Data curation-Supporting, Supervision-Supporting, Writing—review and editing-Supporting), Agnieszka Wroczyńska (Supervision-Supporting, Writing—original draft-Supporting, Writing—review and editing-Supporting), Beata Biernat (Writing—review and editing-Supporting), Katarzyna Sikorska (Supervision-Supporting, Writing—review and editing-Supporting).
